# Analysis of factors influencing health-related quality of life in patients with femoropopliteal atherosclerotic occlusive disease treated with drug-coated balloons 12 months after surgery

**DOI:** 10.3389/fsurg.2025.1657478

**Published:** 2025-11-12

**Authors:** Yan Zhang, Shuangshuang Lu, Weijian Fan, Meng Ye, Ziheng Wu, Zibo Feng, Lianrui Guo, Zhenyu Shi, Xin Fang, Chunshui He, Weihao Shi, Yanpei Cao

**Affiliations:** 1 Nursing Department, Huashan Hospital of Fudan University, Shanghai, China; 2 School of Nursing, Fudan University, Shanghai, China; 3 Department of Vascular Surgery, Huashan Hospital of Fudan University, Shanghai, China; 4 Department of Vascular Surgery, Renji Hospital, School of Medicine, Shanghai Jiaotong University, Shanghai, China; 5 Department of Vascular Surgery, The First Affiliated Hospital, Zhejiang University, School of Medicine, Hangzhou, China; 6 Department of Vascular Surgery, Liyuan Hospital Affiliated Tongji Medical Collage of Huazhong University of Science & Technology, Wuhan, China; 7 Department of Vascular Surgery, Xuanwu Hospital, Capital Medical University and Institute of Vascular Surgery, Capital Medical University, Beijing, China; 8 Department of Vascular Surgery, Zhongshan Hospital of Fudan University, Shanghai, China; 9 Department of Vascular Surgery, Affiliated Hangzhou First People’s Hospital, Zhejiang University School of Medicine, Hangzhou, China; 10 Department of Vascular Surgery, Hospital of Chengdu University of Traditional Chinese Medicine, Chengdu, China

**Keywords:** femoropopliteal occlusive disease, drug-coated balloons, health-related quality of life, multicenter cross-sectional study, rutherford classification, vascuQoL

## Abstract

**Background:**

Patients with femoropopliteal (FP) occlusive disease encounter considerable obstacles concerning health-related quality of life (HRQoL), which serve as the primary objectives of their interventions. While Drug-Coated Balloons (DCBs) present potential advantages, they are not constitute definitive cures. There is a paucity of research concerning postoperative HRQoL in these patients. This study evaluates HRQoL 12 months post-DCB treatment and examines influencing risk factors through a multicenter cross-sectional study.

**Methods:**

This retrospective, multicenter study involved 1012 patients with FP occlusive disease who underwent DCB at 8 vascular centers from August 2021 to December 2023. Data on initial hospitalizations and 12-month follow-up were gathered, and logistic regression was utilized to examine the influencing factors.

**Results:**

According to the median HRQoL at 12 months postoperatively, patients were categorized into low (*N* = 503) and high (*N* = 509) HRQoL groups. Significant differences were found in several variables such as renal insufficiency, calcification degree and TLR incidence (*P* < 0.05), while intervention approach (*P* = 0.781), DCB diameter (*P* = 0.301) and DCB length (*P* = 0.368) showed no significant differences. Logistic regression demonstrated that arterial calcification (OR = 0.33–0.44, *P* < 0.001), postoperative Rutherford classification (grade 1–6, OR = 0.0000 to 0.0367, *P* < 0.001), the Rutherford classification progression within 12 months (OR = 9.53, *P* < 0.001), and target lesion revascularization (TLR) occurrence (OR = 0.09, *P* = 0.011) were significantly linked to HRQoL at 12 months postoperatively, with no significant differences for other factors.

**Conclusions:**

Overall, the Rutherford classification progression over 12 months was significantly positively linked to HRQoL 12 months postoperatively. Conversely, HRQoL was notably diminished in patients who exhibited arterial calcification, elevated postoperative Rutherford classification, and experienced TLR. Nevertheless, intervention approach, DCB length and diameter had no significant relationship to postoperative HRQoL.

## 1 Introduction

Peripheral Artery Disease (PAD) is a chronic, progressive disease primarily caused by atherosclerosis, predominantly impacting middle-aged and elderly individuals (1), with Femoropopliteal (FP) occlusive disease being the most prevalent (2). In 2019, approximately 113 million individuals globally were afflicted with PAD (3). The initial symptoms of the disease are often atypical or absent, but as the disease progresses, patients are likely to experience chronic limb ischemia symptoms, such as pain, intermittent claudication and ulcers (4, 5). A systematic review indicates that PAD, whether symptomatic or not, is linked to increased cardiovascular morbidity and mortality, in conjunction with a deterioration in health-related quality of life (HRQoL) (6). As a prevalent global health issue characterized by its chronic nature and propensity for recurrence, FP occlusive disease significantly impacts the physical and mental well-being of individuals who are afflicted.

Multiple guidelines recommend endovascular intervention as the preferred approach for femoropopliteal (FP) occlusive disease (7). Standard percutaneous transluminal angioplasty (PTA) and arterial stenting have been commonly employed for the treatment of FP occlusive disease (8). However, in-stent restenosis (ISR) may arise post-procedure due to factors such as elastic recoil of the vessel wall and the irritation from the stent (9). Drug-Coated Balloons (DCBs), which are balloons coated with pharmacological agents that inhibit endothelial cell proliferation, have seen a growing application in the treatment of FP occlusive disease in recent years (10). Numerous studies have indicated that DCBs enhance patency rates and decrease the frequency of target lesion revascularization (TLR) (11–16). Nevertheless, despite the positive outcomes associated with DCBs for patients with PAD, the challenge of restenosis following DCB persists as a significant concern (11, 17, 18).

In contrast to other cardiovascular diseases, the management of peripheral artery disease (PAD) primarily emphasizes the enhancement of overall health, rather than exclusively prioritizing survival or limb preservation (19). For patients with FP occlusive disease, enhancements in HRQoL, including pain alleviation and enhanced walking capacity, represent the primary objectives of their interventions. These improvements also serve as critical metrics for patients to evaluate the effectiveness of their medical intervention. According to the European Society for Vascular Surgery (ESVS) 2024 Clinical Practice Guidelines (1), emphasis on HRQoL following endovascular therapy in patients with PAD has been advocated as one of the recommended directions for future research in lower extremity arterial disease. However, the majority of existing studies have not prioritized HRQoL as a primary outcome measure following DCB treatment for FP occlusive disease. Furthermore, there is a notable lack of studies that specifically examine the determinants influencing HRQoL following DCB treatment among patients with FP occlusive disease. Consequently, the objective of this study was to assess HRQoL in individuals with FP occlusive disease who received DCB therapy, evaluated at 12 months postoperatively, utilizing a multicenter cross-sectional design. Additionally, the study sought to conduct a preliminary investigation into the risk factors impacting postoperative HRQoL.

## 2 Methods and methods

### 2.1 Study design

This retrospective study, conducted across various centers at one time point, was designed to identify factors affecting HRQoL of patients suffer from FP occlusive disease who underwent DCB at 12 months postoperatively, and the dependent variable was patients’ HRQoL at 12 months postoperatively.

### 2.2 Setting and study population

Patients diagnosed with FP occlusive disease who received DCB treatment were chosen using a convenience sampling method and were admitted to the Department of Vascular Surgery at 8 vascular centers in China between August 2021 and December 2023. The study data were authorized by the respective hospital. The types of variables included in the study were systematically collected across all centers. The surgical techniques employed across all centers are standardized, which were vascular interventions, including DCB, Debulking procedures, and stent implantation when necessary.

The following criteria were used for inclusion: individuals aged 18 years and older with a projected survival time exceeding 24 months; a Rutherford Classification ranging from 1 to 6; evidence from computed tomography angiography (CTA) indicating the occurrence of an occlusive lesion in the FP artery; inclusion of DCB in the proposed endovascular treatment strategy; for patients presenting with concurrent aortoiliac artery disease, endovascular luminal reconstruction was conducted to achieve flow recanalization with residual stenosis not exceeding 50%; and the presence of at least one healthy infrapopliteal outflow tract measuring 10 cm or more, which is continuous with the infrapopliteal artery either directly or through lateral branches, with a healthy outflow tract defined as having no more than 50% stenosis upon visual inspection; individuals administered double anti platelet therapy (DAPT).

The following criteria were used for exclusion: individuals presenting with concurrent severe insufficiency of cardiac, cerebral, renal, or other critical organ systems; individuals with documented allergies or hypersensitivity to contrast agents, heparin, aspirin, and other anticoagulant or antiplatelet therapies; individuals experiencing intraoperative procedural changes, intermediate incisional thrombectomy, and/or hybridization; individuals’ important data has been lost, such as the basic data, demographics and follow-up information; and individuals enrolled in other research studies at that time. The study relied on secondary data without any personal identification details. Informed consent was waived due to the study's retrospective approach.

### 2.3 Study size

Ni at al.,(2020) suggest a sample size 5–10 times greater than the count of independent variables when calculating for influencing factors (20). The study incorporated 32 independent variables and account for a data loss rate of 15%, resulting in a required minimum sample size ranging from 184 to 368 cases.

### 2.4 Variables

The researchers systematically collected data by reviewing the hospital case system, which included patients’ baseline information, lesion characteristics, treatment information, follow-up information, and HRQoL assessmentsat at both admission and 12 months postoperatively. YZ and WS subsequently organized the data into standardized, pre-agreed data extraction sheets, and any discrepancies were addressed through collaborative discussion.

#### 2.4.1 Basic information and demographics

Baseline data was gathered through a researcher-designed form, developed following a comprehensive review of pertinent literature. The form contained variables involving body mass index (BMI), gender, age, household income, household sizes, smoking, account of chronic ailments [including hyperlipidemia, diabetes mellitus, renal insufficiency, coronary artery disease, hypertension, chronic obstructive pulmonary disease (COPD), and cerebral infarction], previous treatments for PAD, and admission HRQoL.

#### 2.4.2 Lesion characteristics

The lesion characteristics, including preoperative Rutherford classification, preoperative manifestations of acute ischemia, lesion site, the presence of occlusive disease at other sites, the Trans-Atlantic Inter-Society Consensus II (TASC II) classification, lesion length, Vascular calcification (VC), preoperative outflow tract status, stenosis or occlusion, were assessed. One to three days prior to treatment, Rutherford classification and the presence of acute ischemic manifestations of each patient were documented.

Computed tomography (CT) scans were employed to measure the calcification traits of the occluded FP artery occlusion. VC was categorized as follows: mild calcification was characterized by calcification appearing on a single side of the artery, measuring under 5 cm; moderate calcification was identified by calcification on both sides of the artery, measuring between 5 and 10 cm in length; severe calcification was defined by the presence of calcification on both sides of the artery, exceeding 10 cm in length.

Prior to treatment, angiography was utilized to examine lesion site, the existence of occlusive disease at other sites, lesion length, and the infrapopliteal outflow tract status. Lower extremity arterial lesions were classified according to TASC II based on imaging findings (21). To evaluate the below-knee outflow tracts, a runoff score was calculated using the angiographic observations from anterior tibial, posterior tibial, and peroneal arteries (22), Each infrapopliteal artery was independently evaluated on a scale from 0 to 3 (0 = normal artery with minimal lesion; 1 = artery open with stenosis ranging from 20% to 49% of its diameter; 2 = artery open with narrowing ranging from 50% to 99% of its diameter; 2.5 = artery blocked by a clot with less than 50% of its height; 3 = an artery with a thrombus exceeding 50% of its height). The tibial runoff score was calculated by adding 1 to the sum of the three values. A score below 7 was classified as good, while a score of 7 or higher was classified as poor.

#### 2.4.3 Treatment and follow-up information

The treatment information encompassed variables such as endovascular intervention approach, the diameter and length of DCB, and the postoperative Rutherford classification. The approachs encompassed DCB monotherapy, DCB in conjunction with remedial stenting, DCB with stent coverage, directional atherectomy (DA) combined with DCB, and laser atherectomy (LA) combined with DCB.

Follow-up information assessments involved monitoring the consistency of antithrombotic medication administration, the progression of the Rutherford Classification within 12 month, Limb retention status (including minor or major amputation) and TLR status at 12 months following the treatment.

#### 2.4.4 HRQoL

In this research, HRQoL in patients suffering from FP occlusive disease was evaluated at the time of admission and subsequently re-evaluated 12 months following surgical intervention. HRQoL at 12 months post-treatment with DCB was designated as the dependent variable. The assessment utilized the Chinese version visual Vascular Quality of Life Questionnaire (VascuQoL). VascuQoL, initially created by Morgan et al. in 2001, was a specifical tool for evaluating quality of life in PAD (23), and was recommended by clinical guidelines for evaluating HRQoL in patients suffering from PAD (1, 4). The questionnaire comprised 5 dimensions and 25 items: pain (4 items), activity (8 items), symptoms (4 items), socialization (2 items), and mood (7 items). Scores varied between 1 and 7 for each item, with higher scores indicating enhanced HRQoL. In 2023, Zhu Jingpu and colleagues adapted and visualized the VascuQoL for cross-cultural contexts (24). They modified the response options of the original VascuQoL to a graded underlined format and incorporated facial expression —frustrated, smiling, and laughing—as rating anchors corresponding to scores of 1, 4, and 7, respectively.

Exhibiting by a Cronbach's *α* of 0.969, a KMO value of 0.783, and a Bartlett's test of sphericity at 1040.15 (*p* < 0.01), the Chinese version visual VascuQoL proved to be highly reliable and valid. Consequently, this study utilized the Chinese version visual VascuQoL to evaluate HRQoL in patients.

#### 2.4.5 Statistical methods

This study employed SPSS27.0 for statistical data analysis software to analyze the data. At present, there is no standardized grading system for VascuQoL. Therefore, this paper categorized patients into two groups—those with a low HRQoL and those with a high HRQoL—based on the median HRQoL score at 12 months postoperatively. The analysis was conducted on these two groups. For continuous variables following a normal distribution, data were expressed as mean ± standard deviation, with univariable analysis of variance (ANOVA) used for comparisons across multiple groups. For continuous variables not following a normal distribution, data were expressed as median (quartiles) [M(P25,P75)], and Intergroup comparisons were conducted using a non-parametric rank-sum test. Categorical data were reported as frequencies and percentages [cases (%)], with group comparisons conducted using either the chi-square test or Fisher's exact probability method, contingent upon the data characteristics. In the logistic regression analysis, potential risk factors were detected by initially performing a univariable logistic regression., using HRQoL at 12 months post-DCB treatment as the dependent variable. Based on statistically significant results in variance analysis (*P* < 0.05), independent variables were selected. To mitigate the risk of omitting important clinical variables, all items with *P* < 0.10 in the results of univariable logistic regression analysis were subsequently incorporated as independent variables in the multivariable logistic regression to conduct further analysis.

## 3 Results

### 3.1 Patient characteristics and difference analysis results

**Figure 1:**
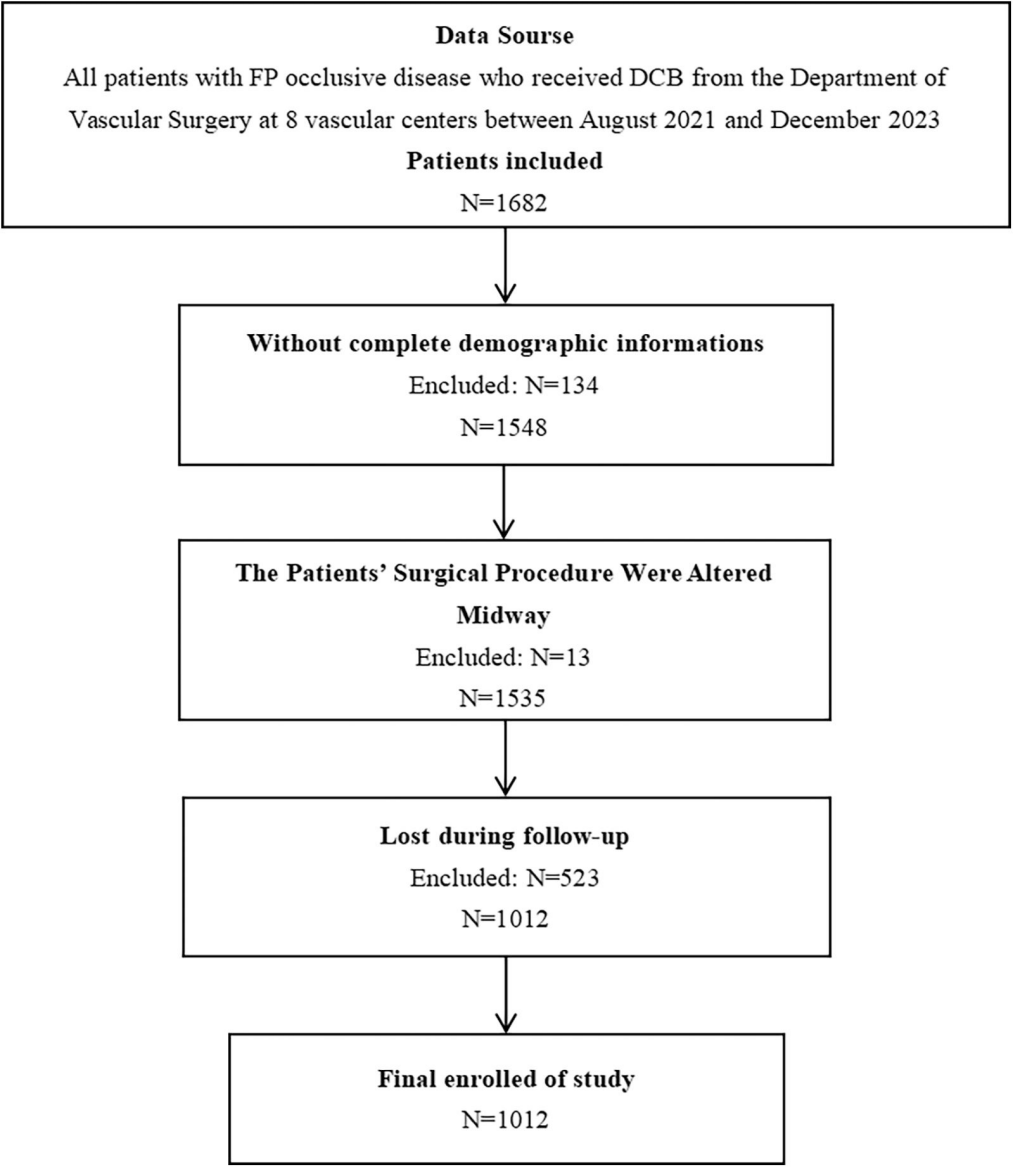
Participant flowchart.

According to the established criteria for inclusion and exclusion, this study encompassed 1,012 patients diagnosed with FP occlusive disease, comprising 727 males (71.8%) and 285 females (28.2%), as shown in Figure 1. The median HRQoL score at 12 months following treatment with DCB was 141. Those with scores below 141 were classified as the low HRQoL group, comprising 503 patients (49.70%), while those with scores of 141 or higher were classified as having a high HRQoL group, comprising 509 (50.30%). The HRQoL scores at 12 months postoperatively served as a binary dependent variable. A detailed comparison of the patients’ characteristics was shown in Supplementary Table S1.

Statistically significant differences (*P* < 0.05), as detailed in Table 1, in BMI, HRQoL on admission, progression of the Rutherford classification within 12 months, gender, age, household size, diabetes mellitus, renal insufficiency, preoperative Rutherford classification, preoperative runoff scores, lesion site, degree of calcification, the presence of occlusive disease at other sites, postoperative Rutherford classification, the presence of TLR and amputation between the two groups. Conversely, DCB length, DCB diameter, TASC classification, monthly household income, smoking, hypertension, hyperlipidemia, coronary artery disease, cerebral infarction, COPD, history of PAD treatment, preoperative manifestations of acute ischemia, stenosis or occlusion, lesion length, endovascular intervention approach and the presence of regular antithrombotic medication were not statistically significant (*P* > 0.05).

**Table 1:** Patients’ characteristics with significantly difference between the two groups.

Variables	All (*n* = 1,012)	Low level group (*n* = 503)	High level group (*n* = 509)	*P*
Age, years	70.00 (65.00, 77.00)	71.00 (65.00,79.00)	70.00 (64.00, 76.00)	<0.001
BMI	25.33 (22.32, 28.91)	25.95 (22.81, 29.37)	24.57 (21.88, 28.37)	<0.001
Admission HRQoL	67.25 (51.00, 81.00)	63.00 (47.50, 79.00)	72.00 (55.00, 84.00)	<0.001
Progression of The Rutherford Classification within 12 months	1.00 (0.00, 2.00)	0.00(-1.00, 2.00)	1.00 (0.00, 3.00)	<0.001
Gender				<0.001
Male	727 (71.80%)	334 (66.40%)	393 (77.21%)	
Female	285 (28.20%)	169 (33.60%)	116 (22.79%)	
Household size				0.013
≤2	324 (32.00%)	148 (29.42%)	176 (34.58%)	
3	184 (18.20%)	82 (16.30%)	102 (20.04%)	
4	192 (19.00%)	95 (18.89%)	97 (19.06%)	
≥5	312 (30.80%)	178 (35.39%)	134 (26.33%)	
Diabetes mellitus				0.024
No	381 (37.60%)	172 (34.19%)	209 (41.06%)	
Yes	631 (62.40%)	331 (65.81%)	300 (58.94%)	
Renal insufficiency				0.006
No	917 (90.60%)	443 (88.07%)	474 (93.12%)	
Yes	95 (9.40%)	60 (11.93%)	35 (6.88%)	
Preoperative Rutherford classification				<0.001
1	16 (1.60%)	5 (0.99%)	11 (2.16%)	
2	161 (15.90%)	67 (13.32%)	94 (18.47%)	
3	412 (40.70%)	184 (36.58%)	228 (44.79%)	
4	110 (10.90%)	62 (12.33%)	48 (9.43%)	
5	287 (28.40%)	168 (33.40%)	119 (23.38%)	
6	26 (2.60%)	17 (3.38%)	9 (1.77%)	
Preoperative runoff scores				0.002
<7	674 (66.60%)	312 (62.03%)	362 (71.12%)	
≥7	338 (33.40%)	191 (37.97%)	147 (28.88%)	
Lesion site				0.016
Involvement of femoral arteries	475 (46.90%)	216 (42.94%)	259 (50.88%)	
Involvement of popliteal arteries	70 (6.90%)	32 (6.36%)	38 (7.47%)	
Involvement of femoral and popliteal arteries	467 (46.10%)	255 (50.70%)	212 (41.65%)	
Degree of calcification				0.025
Without calcification	193 (19.10%)	79 (15.71%)	114 (22.40%)	
Mild calcification	350 (34.60%)	191 (37.97%)	159 (31.24%)	
Moderate calcification	303 (29.90%)	149 (29.62%)	154 (30.26%)	
Severe calcification	166 (16.40%)	84 (16.70%)	82 (16.11%)	
Occlusive disease at other sites				0.032
No other lesions	385 (38.00%)	174 (34.59%)	211 (41.45%)	
Combined with aortoiliac artery segment	478 (47.20%)	259 (51.49%)	219 (43.03%)	
Combined with infrapopliteal artery segment	111 (11.00%)	49 (9.74%)	62 (12.18%)	
Combined with aortoiliac and infrapopliteal artery segment	38 (3.80%)	21 (4.17%)	17 (3.34%)	
Postoperative Rutherford classification				<0.001
0	306 (30.20%)	108 (21.47%)	198 (38.90%)	
1	196 (19.40%)	109 (21.67%)	87 (17.09%)	
2	173 (17.10%)	90 (17.89%)	83 (16.31%)	
3	59 (5.80%)	28 (5.57%)	31 (6.09%)	
4	10 (1.00%)	6 (1.19%)	4 (0.79%)	
5	243 (24.0%)	143 (28.43%)	100 (19.65%)	
6	25 (2.50%)	19 (3.78%)	6 (1.18%)	
Presence of TLR				<0.001
No	994 (98.20%)	487 (96.82%)	507 (99.61%)	
Yes	18 (1.80%)	16 (3.18%)	2 (0.39%)	
Presence of amputation				<0.001
No	995 (98.32%)	486 (96.62%)	509 (100.00%)	
Minor amputation	12 (1.19%)	12 (2.39%)	0 (0.00%)	
Major amputation	5 (0.49%)	5 (0.99%)	0 (0.00%)	

For continuous variables following a normal distribution, data were expressed as mean ± standard deviation; for continuous variables not following a normal distribution, data were expressed as median (quartiles) [M(P25,P75)].

BMI, body mass index; HRQoL, health-related quality of life; TLR, target lesion revascularization; P, *P*-value.

### 3.2 Univariable logistic regression analysis

#### 3.2.1 Basic information and demographics

The results of univariable logistic regression analysis indicated that multiple independent variables exerted a statistically significant impact on patients’ HRQoL at 12 months following treatment with DCB (*P* < 0.05), as depicted in Table 2. In the analysis of variables in Base information and demographics, female patients had a lower likelihood of higher HRQoL scores, with an Odds Ratio (OR) of 0.58 compared to males [95% confidence interval (CI): 0.44–0.77, *P* < 0.001]. The OR for HRQoL scores decreased by 2% for each additional year of age (95% CI: 0.97–0.99, *P* = 0.003) and by 3% for every unit rise in BMI (95% CI: 0.95–0.99, *P* = 0.014), suggesting that both advancing age and elevated BMI may be correlated with diminished HRQoL. Larger household size was linked to better HRQoL, with an OR of 0.37 for those with five or more household members compared to those with two or fewer (95% CI: 0.46–0.87, *P* = 0.004). The OR for HRQoL scores in diabetic patients was 25% lower compared to non-diabetic patients (95% CI: 0.58–0.96, *P* = 0.024). Similarly, the OR for HRQoL scores in patients with renal insufficiency was 0.45% lower than in those without renal insufficiency (95% CI: 0.35–0.84, *P* = 0.006). These observations imply that diabetes and renal insufficiency may serve as risk factors adversely affecting HRQoL. Additionally, each 1-point increase in admission HRQoL correlated with a 1% rise in OR for post-surgery HRQoL (95% CI: 1.01–1.02, *P* < 0.001), revealing that patients exhibiting a higher HRQoL upon admission may experience an elevated HRQoL following surgical intervention.

**Table 2:** Univariable logistic regression analysis result.

Variables	β	S.E	OR(95% CI)	*P*
Age	−0.03	0.01	0.98 (0.97–0.99)	0.003
BMI	−0.03	0.01	0.97 (0.95–0.99)	0.014
Admission HRQoL	0.01	0.00	1.01 (1.01–1.02)	<0.001
Progression of the Rutherford classification within 12 months	0.24	0.03	1.32 (1.23–1.41)	<0.001
Gender				
Male				
Female	−0.54	0.14	0.58 (0.44–0.77)	<0.001
Household size				
≤2				
3	0.04	0.19	1.05 (0.73–1.50)	0.808
4	−0.15	0.18	0.86 (0.60–1.23)	0.403
≥5	−0.46	0.16	0.63 (0.46–0.87)	0.004
Diabetes mellitus				
No				
Yes	−0.29	0.13	0.75 (0.58–0.96)	0.024
Renal insufficiency				
No				
Yes	−0.61	0.22	0.55 (0.35–0.84)	0.006
Preoperative Rutherford classification				
1				
2	−0.45	0.56	0.64 (0.21–1.92)	0.424
3	−0.57	0.55	0.56 (0.19–1.65)	0.295
4	−1.04	0.57	0.35 (0.11–1.08)	0.068
5	−1.13	0.55	0.32 (0.11–0.95)	0.040
6	−1.42	0.68	0.24 (0.06–0.91)	0.036
Preoperative runoff scores				
<7				
≥7	−0.41	0.13	0.66 (0.51–0.86)	0.002
Lesion site				
Involvement of femoral arteries				
Involvement of popliteal arteries	−0.01	0.26	0.99 (0.60–1.64)	0.970
Involvement of femoral and popliteal arteries	−0.37	0.13	0.69 (0.54–0.90)	0.005
Degree of calcification				
Without calcification				
Mild calcification	−0.55	0.18	0.58 (0.40–0.82)	0.002
Moderate calcification	−0.33	0.19	0.72 (0.50–1.03)	0.073
Severe calcification	−0.39	0.21	0.68 (0.45–1.03)	0.067
Occlusive disease at other sites				
No other lesions				
Combined with aortoiliac artery segment	0.04	0.22	1.04 (0.68–1.60)	0.845
Combined with infrapopliteal artery segment	−0.36	0.14	0.70 (0.53–0.91)	0.009
Combined with aortoiliac and infrapopliteal artery segment	−0.40	0.34	0.67 (0.34–1.30)	0.237
Postoperative Rutherford classification				
0				
1	−0.83	0.19	0.44 (0.30–0.63)	<0.001
2	−0.69	0.19	0.50 (0.34–0.74)	<0.001
3	−0.50	0.29	0.60 (0.34–1.06)	0.079
4	−1.01	0.66	0.36 (0.10–1.32)	0.123
5	−0.96	0.18	0.38 (0.27–0.54)	<0.001
6	−1.76	0.48	0.17 (0.07–0.44)	<0.001
Presence of TLR				
No				
Yes	−2.12	0.75	0.12 (0.03–0.52)	0.005
Presence of amputation				
No				
Minor amputation	−15.61	650.87	0.00 (0.00–Inf)	0.981
Major amputation	−15.61	420.14	0.00 (0.00–Inf)	0.970

BMI, body mass index; HRQoL, health-related quality of life; TLR, target lesion revascularization; P, *P*-value; β, beta coefficient; S.E, standard error; OR, odds ratio; 95% CI, 95% confidence interval.

#### 3.2.2 Lesion characteristics

In the analysis of variables in lesion characteristics, the OR for HRQoL scores in patients with a preoperative Rutherford classification of grade 6 was 0.24 compared to those with grade 1 (95% CI: 0.06–0.91, *P* = 0.036), which indicates that patients with a higher severity of illness preoperatively experienced significantly lower HRQoL scores. Moreover, an OR of 0.66 was found for individuals with a preoperative runoff score of ≥7 (95% CI: 0.51–0.86, *P* = 0.002), revealing that poorer status of below-knee outflow tracts may be associated with reduced HRQoL. The OR for patients exhibiting mild calcification was 0.58 (95% CI: 0.40–0.82, *P* = 0.002), indicating that the extent of calcification may have an impact on HRQoL. Furthermore, the OR for HRQoL scores was 31% lower in individuals with lesions affecting both the femoral and popliteal arteries (95% CI: 0.54–0.90, *P* = 0.005) and 30% lower in those with combined infrapopliteal arterial occlusive lesions (95% CI: 0.53–0.91, *P* = 0.009), demonstrating that HRQoL may be diminished in patients with a greater number of lesion sites.

#### 3.2.3 Treatment and follow-up information

In the analysis of variables in treatment information and postoperative follow-up, patients with several postoperative Rutherford classification grades (grades 1, 2, 5 and 6) demonstrated a significantly lower OR for HRQoL scores, ranging from 0.17 to 0.44 (*P* < 0.001). Moreover, the OR for HRQoL scores increased by 27% for each additional level of improvement in the Rutherford classification, indicating that patients exhibiting a greater degree of improvement in the Rutherford classification tend to experience an enhanced HRQoL (95% CI: 1.19–1.36, *P* < 0.001). Furthermore, the OR for patients who underwent TLR was only 0.12 (95% CI: 0.03–0.52, *P* = 0.005), signifying that TLR events were associated with a markedly diminished HRQoL scores. In contrast, the multivariable model showed that statistical significance was not achieved by the presence of amputation (*p* > 0.1).

### 3.3 Multivariable logistic regression analysis

After adjusting for confounders in the multivariable logistic regression analysis, as detailed in Table 3, it was found that degree of calcification, postoperative Rutherford classification, progression of the Rutherford classification within a 12-month period and TLR status, independently influenced patients’ HRQoL scores at 12 months postoperatively (*P* < 0.05). Notably, the OR for patients with mild calcification, moderate calcification and severe calcification were 0.36 (95% CI 0.20–0.63, *P* < 0.001), 0.33 (95% CI 0.18–0.60, *P* < 0.001) and 0.44 (95% CI 0.23–0.85, *P* = 0.015), respectively. These findings suggested that vascular calcification is correlated with a reduced benefit in HRQoL scores. Furthermore, the OR for HRQoL scores among patients classified as postoperative Rutherford class 1, 2, 3 and 4 were found to be 3.67% (95% CI 0.0192–0.0701, *P* < 0.001), 0.49% (95% CI 0.0021–0.0114, *P* < 0.001), 0.09% (95% CI 0.0003–0.0032, *P* < 0.001) and 0.02% (95% CI 0.0000–0.0019, *P* < 0.001) lower than those of class 0, respectively, while the OR for Rutherford class 5 and 6 was so minimal that it was represented as 0.0000 (95% CI 0.0000–0.0001, *P* < 0.001).These findings indicated that a higher postoperative classification was significantly associated with a diminished advantage in HRQoL scores. Additionally, for each 1-point rise in the Rutherford classification over 12 months, the OR of HRQoL scores increased to 9.53 times (95% CI 6.91–13.14, *P* < 0.001), which suggested that a greater degree of improvement in the Rutherford classification was more likely to lead to higher HRQoL postoperatively. The OR for patients who experienced TLR was 0.09 (95% CI 0.01–0.57, *P* = 0.011), further corroborating the association between TLR events and significantly lower HRQoL scores. In contrast, the multivariable model showed that statistical significance was not achieved by other variables, such as gender, renal insufficiency and admission HRQoL (*P* > 0.05), indicating that their independent effects on the HRQoL scores were no longer significant after adjustment for confounders.

**Table 3:** Multivariable logistic regression analysis result.

Variables	β	S.E	OR(95% CI)	*P*
Age	−0.02	0.01	0.98 (0.95–1.01)	0.180
BMI	0.03	0.02	1.03 (0.99–1.08)	0.149
Admission HRQoL	0.00	0.00	1.00 (1.00–1.01)	0.384
Progression of The Rutherford Classification within 12 months	2.25	0.16	9.53 (6.91–13.14)	<0.001
Gender				
Male				
Female	−0.13	0.23	0.87 (0.55–1.38)	0.566
Household size				
≤2				
3	0.46	0.29	1.58 (0.89–2.80)	0.120
4	0.41	0.30	1.51 (0.84–2.70)	0.168
≥5	0.06	0.26	1.06 (0.63–1.77)	0.829
Diabetes mellitus				
No				
Yes	−0.07	0.20	0.93 (0.63–1.37)	0.723
Renal insufficiency				
No				
Yes	−0.36	0.35	0.70 (0.35–1.39)	0.308
Preoperative Rutherford classification				
1				
2	−1.31	0.78	0.27 (0.06–1.24)	0.092
3	−1.10	0.76	0.33 (0.08–1.46)	0.145
4	−1.41	0.81	0.24 (0.05–1.19)	0.081
5	−0.94	0.88	0.39 (0.07–2.19)	0.286
6	−0.54	1.54	0.58 (0.03–11.82)	0.726
Preoperative runoff scores				
<7				
≥7	−0.12	0.26	0.88 (0.54–1.46)	0.628
Lesion site				
Involvement of femoral arteries				
Involvement of popliteal arteries	0.54	0.42	1.71 (0.75–3.90)	0.202
Involvement of femoral and popliteal arteries	0.14	0.20	1.15 (0.77–1.71)	0.485
Degree of calcification				
Without calcification				
Mild calcification	−1.02	0.29	0.36 (0.20–0.63)	<0.001
Moderate calcification	−1.10	0.30	0.33 (0.18–0.60)	<0.001
Severe calcification	−0.82	0.34	0.44 (0.23–0.85)	0.015
Occlusive disease at other sites				
No other lesions				
Combined with aortoiliac artery segment	0.28	0.31	1.33 (0.73–2.43)	0.354
Combined with infrapopliteal artery segment	0.24	0.26	1.27 (0.77–2.10)	0.345
Combined with aortoiliac and infrapopliteal artery segment	−0.22	0.52	0.80 (0.29–2.21)	0.667
Postoperative Rutherford classification				
0				
1	−3.31	0.33	0.0367 (0.0192–0.0701)	<0.001
2	−5.32	0.44	0.0049 (0.0021–0.0114)	<0.001
3	−7.00	0.64	0.0009 (0.0003–0.0032)	<0.001
4	−8.64	1.21	0.0002 (0.0000–0.0019)	<0.001
5	−11.06	0.93	0.0000 (0.0000–0.0001)	<0.001
6	−11.97	1.56	0.0000 (0.0000–0.0001)	<0.001
Presence of TLR				
No				
Yes	−2.44	0.96	0.09 (0.01–0.57)	0.011

BMI, body mass index; HRQoL, health-related quality of life; TLR, target lesion revascularization; P, *P*-value; β, beta coefficient; S.E, standard error; OR, odds ratio; 95% CI, 95% confidence interval.

## 4 Discussion

HRQoL is frequently defined as a concept that relates to the health aspect of quality of life, emphasizing how illness and treatment affect disability, daily activities, and the role of health status in one's capacity to experience a contented life. HRQoL evaluates quality of life by accounting for the effect of illness, injury, medical intervention, and policy on impairment, functional status, perception, and opportunities (25, 26). HRQoL generally encompasses physical, psychological, and social support dimensions, and comprehending HRQoL is critical for advancing symptom relief, patient treatment, and recovery. Patient self-reported HRQoL can offers valuable insights into the suitability and potential improvements of specific treatment modalities. As highlighted in the introduction, for many patients with FP occlusive disease, alterations in HRQoL are the primary motivation for seeking medical intervention, and serve as the principal metric for assessing procedural efficacy. Consequently, this study aims to propose a comprehensive large-scale study to preliminarily investigate the determinants affecting postoperative HRQoL in patients with FP occlusive disease undergoing DCB.

### 4.1 Vascular calcification

The findings of logistic regression analysis revealed that indicated that variables such as lesion site, DCB length, DCB diameter, and preoperative outflow tract condition did not exhibit a statistically significant association with HRQoL at 12 months postoperatively (*P* > 0.05). Conversely, degree of calcification and postoperative Rutherford classification demonstrated a significant association with HRQoL at 12 months following surgery. Vascular calcification, particularly medial arterial calcification, emerges as a critical risk factor for PAD and may compromise the effectiveness of Endovascular intervention (27, 28). Furthermore, VC adversely impacts the efficacy of stenting in standard PTA and elevates the risk of TLR (29, 30). The study carried out by Soga and colleagues demonstrated that DCB treatment for FP occlusive disease with mild calcification was associated with improved vascular patency; however, its effectiveness was reduced in patients with severe calcification (31). In contrast, the multivariate logistic regression analysis presented in the current study revealed that the OR for HRQoL scores in patients with mild calcification (OR = 0.36, *P* < 0.001) and moderate calcification (OR = 0.33, *P* < 0.001) was lower than that those with severe calcification (OR = 0.44, *P* < 0.001), which indicated that patients with mild or moderate calcification tend to experience a lower HRQoL at 12 months postoperatively, in comparison to severe calcification. This disparity could be explained by the fact that patients with severely calcified lesions in this research were potentially addressed using DA or LA in conjunction with DCB. Numerous studies have substantiated the effectiveness of these interventions for managing patients with severe calcification over the intermediate and long term (11, 12, 32), thus leading to a higher HRQoL at 12 months postoperatively among individuals with moderate and severe calcifications.

### 4.2 Postoperative rutherford classification and its progression

The results of this study suggested that individuals with a elevated postoperative Rutherford classification exhibited a comparatively lower HRQoL at 12 months. As the classification level increases, the HRQoL correspondingly decreased. The odds ratio for Rutherford classes 5 and 6 was negligible, being reported as 0.0000 (95% CI 0.0000–0.0001, *P* < 0.001). Meanwhile, as the Rutherford classification increases within 12 months, there was a corresponding significant improvement in the patient's HRQoL (OR = 9.53, 95% CI 6.91–13.14, *P* < 0.001). In brief, the aforementioned data collectively suggest a strong correlation between the R grade and patients’ HRQoL. The Rutherford classification is a widely utilized system for staging and grading patients with PAD based on clinical symptoms. An higher grading level within this system is associated with more severe ischemic symptoms in the affected limbs, including intermittent claudication and pain (33). The extent to which postoperative ischemic conditions are effectively ameliorated significantly influences the long-term HRQoL following surgery. Ambulatory capability constitutes a critical aspect of HRQoL, because adequate walking ability and the absence of pain are essential prerequisites for patients to fully engage in life. Alterations in the Rutherford classification during follow-up assessments can serve as direct indicators of enhancements in patients’ walking ability and pain levels.

### 4.3 The occurrence of TLR

Within one year, 18 patients (1.77%) underwent TLR, including 16 patients whose HRQoL scores were below the median of 141 at the 12-month mark. Multivariable logistic regression analysis revealed a significant negative correlation between the occurrence of TLR and HRQoL at 12 months postoperatively (OR = 0.11, *P* = 0.004). Interviews with these patients indicated that the primary reason for hospital readmission for TLR was the recurrence of lower extremity ischemic symptoms, such as coldness, pain, and intermittent claudication, which adversely affected their mobility and daily activities. Furthermore, TLR may induce concerns regarding disease prognosis among patients and impose economic burdens, which can adversely affect patients’ mental health and overall HRQoL.

### 4.4 Limitations

The present research is a retrospective, multicenter cross-sectional investigation, serving as a preliminary exploratory analysis. Convenience sampling was employed to select participants, prioritizing the study's feasibility. Consequently, it is essential to recognize that the findings may may not be representative, thereby limiting the generalizability of the conclusions compared to studies using probability sampling methods. To enhance the validity of these findings, future research should incorporate sampling methods such as simple random sampling or stratified sampling, and employ designs like cohort studies or randomized controlled trials.

The variables examined in this study were derived from patients’ baseline characteristics and treatment-related information, excluding laboratory indices. However, numerous studies have demonstrated that certain laboratory markers significantly influence postoperative restenosis and HRQoL in patients. A prospective observational study by Wachsmann-Maga et al. on patients with PAD identified a link between quality of life changes and markers of vascular inflammation including leukotrienes (LTE4) and thromboxane B2 (TXB2) (34). Additionally, TAO and colleagues performed a retrospective study to investigate the link between postoperative restenosis and anemia in 91 patients with FP occlusive disease who underwent DCB angioplasty (35). Their findings identified anemia (*P* = 0.030) as a risk factor for postoperative restenosis in this patient cohort. Consequently, future research should incorporate laboratory indicators to further explore and validate the relationships between additional variables and HRQoL outcomes following DCB treatment in individuals suffering from FP occlusive disease.

This study is a single time-point cross-sectional analysis, but FP occlusive disease is chronic and recurrent. Patients’ walking ability and HRQoL change over time after DCB treatment. For instance, a patient with FP occlusive disease who underwent DCB may exhibit significantly different HRQoL outcomes at 6 months and 12 months postoperatively. Therefore, Collecting data at multiple postoperative points would allow for a longitudinal analysis of HRQoL alterations, which would facilitate a comprehensive understanding of the HRQoL trajectory among patients with varying characteristics, thereby helping to identify factors influencing HRQoL in FP occlusive disease patients after DCB.

## 5 Conclusion

In the study, we performed a multicenter cross-sectional analysis of HRQoL at 12 months postoperatively among individuals suffer from FP occlusive disease who underwent DCB angioplasty. We examined the factors influencing HRQoL and found that a higher degree of calcification, a higher postoperative Rutherford classification, and those who experienced TLR exhibited lower postoperative HRQoL. Additionally, the progression of the Rutherford classification within 12 months was significantly positively correlated with HRQoL at 12 months postoperatively. This study represents a preliminary investigation into the risk factors affecting long-term HRQoL following DCB treatment. Future research should seek to validate this study's findings through the implementation of randomized sampling, the expansion and refinement of the variable set, and the assessment of patients at multiple time intervals. This method will enable a more accurate identification of factors affecting long-term HRQoL in patients with FP occlusive disease undergoing DCB treatment. Additionally, such studies will contribute to the enhancement of therapeutic interventions, ultimately aiming to improve patients’ ambulatory capabilities and overall HRQoL.

## Data Availability

The original contributions presented in the study are included in the article/Supplementary Material, further inquiries can be directed to the corresponding author.
