# Supplementary material for: Analysis of factors influencing health-related quality of life in patients with femoropopliteal atherosclerotic occlusive disease treated with drug-coated balloons 12 months after surgery
Source: Front Surg. 2025 Nov 12;12:1657478. doi: 10.3389/fsurg.2025.1657478 (PMC12647069; doi:10.3389/fsurg.2025.1657478)
Supplement: Supplementary file 1 [file Supplementaryfile1.docx]

**Table S1.** Patients’ Characteristics and Difference Analysis

| Variables | All (n = 1012) | Low Level Group  (n = 503) | High Level Group  (n = 509) | P |
| --- | --- | --- | --- | --- |
| Age,years | 70.00 (65.00, 77.00) | 71.00 (65.00,79.00) | 70.00 (64.00, 76.00) | <0.001 |
| BMI | 25.33 (22.32, 28.91) | 25.95 (22.81, 29.37) | 24.57 (21.88, 28.37) | <0.001 |
| Admission HRQoL | 67.25 (51.00, 81.00) | 63.00 (47.50, 79.00) | 72.00 (55.00, 84.00) | <0.001 |
| DCB Length | 22.00 (15.00, 30.00) | 22.00 (15.00, 30.00) | 22.00 (15.00, 30.00) | 0.368 |
| Progression of The Rutherford Classification within 12 months | 1.00(0.00, 2.00) | 0.00(-1.00, 2.00) | 1.00(0.00, 3.00) | <0.001 |
| Gender |  |  |  | <0.001 |
| Male | 727 (71.80%) | 334 (66.40%) | 393 (77.21%) |  |
| Female | 285 (28.20%) | 169 (33.60%) | 116 (22.79%) |  |
| Monthly Household Income |  |  |  | 0.783 |
| <5000 | 147 (14.50%) | 75 (14.91%) | 72 (14.15%) |  |
| 5000~10000 | 494 (48.80%) | 240 (47.71%) | 254 (49.90%) |  |
| >10000 | 371 (36.70%) | 188 (37.38%) | 183 (35.95%) |  |
| Number of Family Members |  |  |  | 0.013 |
| ≤2 | 324 (32.00%) | 148 (29.42%) | 176 (34.58%) |  |
| 3 | 184 (18.20%) | 82 (16.30%) | 102 (20.04%) |  |
| 4 | 192 (19.00%) | 95 (18.89%) | 97 (19.06%) |  |
| ≥5 | 312 (30.80%) | 178 (35.39%) | 134 (26.33%) |  |
| Smoking |  |  |  | 0.117 |
| Never or have quit for more than a year | 652 (64.40%) | 336 (66.80%) | 316 (62.08%) |  |
| Yes | 360 (35.60%) | 167 (33.20%) | 193 (37.92%) |  |
| Hypertension |  |  |  | 0.096 |
| No | 269 (26.60%) | 122 (24.25%) | 147 (28.88%) |  |
| Yes | 743 (73.40%) | 381 (75.75%) | 362 (71.12%) |  |
| Diabetes Mellitus |  |  |  | 0.024 |
| No | 381 (37.60%) | 172 (34.19%) | 209 (41.06%) |  |
| Yes | 631 (62.40%) | 331 (65.81%) | 300 (58.94%) |  |
| Hyperlipidemia |  |  |  | 0.562 |
| No | 787 (77.80%) | 395 (78.53%) | 392 (77.01%) |  |
| Yes | 225 (22.20%) | 108 (21.47%) | 117 (22.99%) |  |
| Renal Insufficiency |  |  |  | 0.006 |
| No | 917 (90.60%) | 443 (88.07%) | 474 (93.12%) |  |
| Yes | 95 (9.40%) | 60 (11.93%) | 35 (6.88%) |  |
| Coronary Artery Disease |  |  |  | 0.273 |
| No | 797 (78.80%) | 389 (77.34%) | 408 (80.16%) |  |
| Yes | 215 (21.20%) | 114 (22.66%) | 101 (19.84%) |  |
| Cerebral Infarction History |  |  |  | 0.671 |
| No | 848 (83.80%) | 419 (83.30%) | 429 (84.28%) |  |
| Yes | 164 (16.20%) | 84 (16.70%) | 80 (15.72%) |  |
| COPD |  |  |  | 0.837 |
| No | 661(65.32) | 315(62.62) | 346(67.98) |  |
| Yes | 351(34.68) | 188(37.38) | 163(32.02) |  |
| History of PAD Treatment |  |  |  | 0.074 |
| No | 661 (65.30%) | 315 (62.62%) | 346 (67.98%) |  |
| Yes | 351 (34.70%) | 188 (37.38%) | 163 (32.02%) |  |
| Preoperative Manifestations of Acute Ischemia |  |  |  | 0.080 |
| No | 960 (94.90%) | 471 (93.64%) | 489 (96.07%) |  |
| Yes | 52 (5.10%) | 32 (6.36%) | 20 (3.93%) |  |
| Preoperative Rutherford Classification |  |  |  | <0.001 |
| 1 | 16 (1.60%) | 5 (0.99%) | 11 (2.16%) |  |
| 2 | 161 (15.90%) | 67 (13.32%) | 94 (18.47%) |  |
| 3 | 412 (40.70%) | 184 (36.58%) | 228 (44.79%) |  |
| 4 | 110 (10.90%) | 62 (12.33%) | 48 (9.43%) |  |
| 5 | 287 (28.40%) | 168 (33.40%) | 119 (23.38%) |  |
| 6 | 26 (2.60%) | 17 (3.38%) | 9 (1.77%) |  |
| Preoperative Runoff Scores |  |  |  | 0.002 |
| <7 | 674 (66.60%) | 312 (62.03%) | 362 (71.12%) |  |
| ≥7 | 338 (33.40%) | 191 (37.97%) | 147 (28.88%) |  |
| Stenosis or Occlusion |  |  |  | 0.368 |
| Stenosis | 463(45.75) | 223(44.33) | 240(47.15) |  |
| Occlusion | 549(54.25) | 280(55.67) | 269(52.85) |  |
| Lesion Site |  |  |  | 0.016 |
| Involvement of femoral arteries | 475 (46.90%) | 216 (42.94%) | 259 (50.88%) |  |
| Involvement of popliteal arteries | 70 (6.90%) | 32 (6.36%) | 38 (7.47%) |  |
| Involvement of femoral and popliteal arteries | 467 (46.10%) | 255 (50.70%) | 212 (41.65%) |  |
| TASC classification |  |  |  | 0.156 |
| A | 129 (12.70%) | 65 (12.92%) | 64 (12.57%) |  |
| B | 349 (34.50%) | 167 (33.20%) | 182 (35.76%) |  |
| C | 388 (38.30%) | 186 (36.98%) | 202 (39.69%) |  |
| D | 146 (14.40%) | 85 (16.90%) | 61 (11.98%) |  |
| Lesion Length |  |  |  | 0.081 |
| ≤10 cm | 470 (46.40%) | 251 (49.90%) | 219 (43.03%) |  |
| >10cm ,≤20 cm | 278 (27.50%) | 132 (26.24%) | 146 (28.68%) |  |
| >20 cm | 264 (26.10%) | 120 (23.86%) | 144 (28.29%) |  |
| Degree of calcification |  |  |  | 0.025 |
| Without calcification | 193 (19.10%) | 79 (15.71%) | 114 (22.40%) |  |
| Mild calcification | 350 (34.60%) | 191 (37.97%) | 159 (31.24%) |  |
| Moderate calcification | 303 (29.90%) | 149 (29.62%) | 154 (30.26%) |  |
| Severe calcification | 166 (16.40%) | 84 (16.70%) | 82 (16.11%) |  |
| Occlusive Disease at Other Sites |  |  |  | 0.032 |
| No other lesions | 385 (38.00%) | 174 (34.59%) | 211 (41.45%) |  |
| Combined with aortoiliac artery segment | 478 (47.20%) | 259 (51.49%) | 219 (43.03%) |  |
| Combined with infrapopliteal artery segment | 111 (11.00%) | 49 (9.74%) | 62 (12.18%) |  |
| Combined with aortoiliac and infrapopliteal artery segment | 38 (3.80%) | 21 (4.17%) | 17 (3.34%) |  |
| Intervention Approach |  |  |  | 0.781 |
| DCB monotherapy | 662 (65.40%) | 325 (64.61%) | 337 (66.21%) |  |
| DCB in conjunction with remedial stenting | 182 (18.00%) | 97 (19.28%) | 85 (16.70%) |  |
| DCB with stent coverage | 56 (5.50%) | 26 (5.17%) | 30 (5.89%) |  |
| DA combined with DCB | 100 (9.90%) | 48 (9.54%) | 52 (10.22%) |  |
| LA combined with DCB | 12 (1.20%) | 7 (1.39%) | 5 (0.98%) |  |
| DCB diameter |  |  |  | 0.301 |
| <5mm | 228 (22.50%) | 115 (22.86%) | 113 (22.20%) |  |
| ≥5mm, <6mm | 674 (66.60%) | 341 (67.79%) | 333 (65.42%) |  |
| ≥6mm | 110 (10.90%) | 47 (9.34%) | 63 (12.38%) |  |
| Postoperative Rutherford Classification |  |  |  | <0.001 |
| 0 | 306 (30.20%) | 108 (21.47%) | 198 (38.90%) |  |
| 1 | 196 (19.40%) | 109 (21.67%) | 87 (17.09%) |  |
| 2 | 173 (17.10%) | 90 (17.89%) | 83 (16.31%) |  |
| 3 | 59 (5.80%) | 28 (5.57%) | 31 (6.09%) |  |
| 4 | 10 (1.00%) | 6 (1.19%) | 4 (0.79%) |  |
| 5 | 243 (24.0%) | 143 (28.43%) | 100 (19.65%) |  |
| 6 | 25 (2.50%) | 19 (3.78%) | 6 (1.18%) |  |
| Regular Antithrombotic Medication Administration |  |  |  | 0.088 |
| Yes | 934 (92.30%) | 457(90.85%) | 477(93.71%) |  |
| No | 78 (7.70%) | 46(9.15%) | 32(6.29%) |  |
| Presence of TLR |  |  |  | <0.001 |
| No | 994 (98.20%) | 487 (96.82%) | 507 (99.61%) |  |
| Yes | 18 (1.80%) | 16 (3.18%) | 2 (0.39%) |  |
| Presence of Amputation |  |  |  | <0.001 |
| No | 995(98.32%) | 486(96.62%) | 509(100.00%) |  |
| Minor amputation | 12(1.19%) | 12(2.39%) | 0(0.00%) |  |
| Major amputation | 5(0.49%) | 5(0.99%) | 0(0.00%) |  |

For continuous variables following a normal distribution, data were expressed as mean ± standard deviation; for continuous variables not following a normal distribution, data were expressed as median (quartiles) [M(P25,P75)].

Abbreviations: BMI, body mass index; HRQoL, health-related quality of life; COPD, Chronic obstructive pulmonary disease; PAD, Peripheral Artery Disease; TASC, Trans-Atlantic Inter-Society Consensus II; DCB, Drug-Coated Balloon; DA, directional atherectomy; LA, laser atherectomy; cm, centimetre; TLR, target lesion revascularization; P, P-value.
